# Simple acupuncture combined with fluoxetine in the treatment of poststroke depression: A protocol for systematic review and meta-analysis

**DOI:** 10.1097/MD.0000000000024968

**Published:** 2021-03-12

**Authors:** Peng Gong, Xiujuan Ma, Lijing Gao, Jianhao Bi

**Affiliations:** Weihai Municipal Third Hospital (Mental Health Center of Weihai City), Weihai, Shandong Province, China.

**Keywords:** acupuncture, fluoxetine, meta-analysis, poststroke depression, RCT

## Abstract

**Background:**

Poststroke depression is a common secondary mental disorder after stroke, which increases the recurrence rate and mortality rate after stroke and hinders the recovery of function. As a combination therapy, simple acupuncture combined with fluoxetine has achieved good clinical effect, but there is a lack of evidence-based medicine. The purpose of this study is to evaluate the efficacy and safety of acupuncture combined with fluoxetine in the treatment of poststroke depression by meta-analysis.

**Methods:**

Search Chinese and English databases: China national knowledge infrastructure, VP information Chinese Journal Service Platform, Wanfang, the China Biomedical Database, PubMed, Embase, the Cochrane Library, and web of science. A randomized controlled trial of simple acupuncture combined with fluoxetine in the treatment of poststroke depression will be selected. The retrieval time is of the establishment of the database in January 2021. Selected literature is extracted and deleted by 2 researchers, and the quality of the included literature is evaluated. The included literature is analyzed by Meta with RevMan5.3 software.

**Results:**

In this study, the efficacy and safety of acupuncture combined with fluoxetine in the treatment of post-stroke depression are evaluated by Hamilton Depression scale (HAMD) and its reduction rate, Treatment Emergency Symptom Scale, Self-rating Depression Scale, and Activities of Daily living scale.

**Conclusion:**

This study will provide reliable evidence-based evidence for the clinical application of acupuncture combined with fluoxetine in the treatment of post-stroke depression.

**OSF Registration number:**

DOI 10.17605/OSF.IO/5J896.

## 1 Introduction

Stroke is a permanent damage to brain tissue caused by insufficient blood supply to the brain caused by thrombosis, embolism or hemorrhagic events.^[1]^ According to the latest data from the American Heart Association, there are 700,000 people with stroke and 163,000 people died from stroke each year in the United States.^[2]^ Stroke is the fifth leading cause of death and the leading cause of long-term disability.^[3]^

Poststroke depression is a common and serious complication of stroke,^[4]^ which is characterized by depression, loss of interest and loss of pleasure, anxiety, cognitive and sleep disorders, and even suicidal thoughts.^[5,6]^ Poststroke depression not only hinders the recovery of neurological function, but also increases the mortality of the disease by 3 to 4 times.^[7]^ Due to the different time after stroke and the different diagnostic criteria of poststroke depression, the prevalence rate of poststroke depression is between 5% and 67%.^[8]^ The pathogenesis of poststroke depression is different from general depression, which may be caused directly by brain injury or indirectly by depressed mood after injury.^[9]^ At present, it is believed that its pathogenesis includes not only endogenous factors such as neurochemicals and inflammatory cytokines, but also exogenous factors such as sex, age, marriage, social environment, education, life stress and so on.^[10]^

Treatments for poststroke depression include drug and non-drug interventions (such as psychotherapy, rehabilitation), as well as combination therapy.^[11]^ The American Heart Association and the American Stroke Association jointly recommend antidepressants for the treatment of poststroke depression.^[12]^ At present, selective serotonin reuptake inhibitors^[13]^ are commonly used in clinical drug therapy, including fluoxetine, paroxetine, sertraline and so on. Studies have shown that the use of fluoxetine in patients with poststroke depression reduces the score of the Self-Rating Depression Scale more significantly than that of placebo after 8 weeks, which can effectively improve the depressive symptoms.^[14]^ However, long-term use of depression drugs is easy to cause gastrointestinal reactions, aggravate the metabolic burden of liver and kidney function and other side effects.^[15]^ Acupuncture is recommended as an effective supplement to the treatment of depression as recommended by the 2016 American College of Internal Medicine Clinical practice guidelines for Depression.^[16]^ Acupuncture, based on the meridian theory of traditional Chinese medicine and the balance of Yin, Yang, Qi and blood, punctures human acupoints with needles to achieve the purpose of treatment. Clinical trials have proved that acupuncture alone has a significant effect on poststroke depression, even better than antidepressants, and almost no adverse reactions.^[17]^

At present, there are a number of randomized controlled trials.^[18–21]^ The results show that acupuncture combined with fluoxetine can effectively inhibit depression, shorten the effective time of fluoxetine, reduce adverse reactions and improve the recovery of neurological function in patients. However, the sample size of each clinical trial is small, and the literature quality and efficacy have not been systematically evaluated. Therefore, this study plans to systematically evaluate the efficacy and safety of simple acupuncture combined with fluoxetine in the treatment of poststroke depression, and to provide reliable evidence-based basis for the clinical application of simple acupuncture combined with fluoxetine in the treatment of poststroke depression.

## 2 Methods

### 2.1 Protocol register

This protocol of systematic review and meta-analysis has been drafted under the guidance of the preferred reporting items for systematic reviews and meta-analyses. It will be registered in the open science framework (registration number: DOI 10.17605/OSF.IO/5J896).

### 2.2 Ethics

Since all the data used in this study have been published, there is no need to collect personal information, so the approval of the Ethics Committee is not required. In addition, all data will be anonymously analyzed during the review process.

### 2.3 Eligibility criteria

#### 2.3.1 Types of studies

A randomized controlled trial of simple acupuncture combined with fluoxetine in the treatment of poststroke depression was included. The literature language is limited to Chinese and English. Because of the particularity of acupuncture, the blind method is not limited. There is no limit to the publishing area and time.

#### 2.3.2 Research objects

Patients with poststroke depression are clearly diagnosed. The nationality, race, gender and age of the patients included in the study are not limited. There is no limit to the severity of the disease. There is no limit to cerebral hemorrhage or cerebral ischemia.

#### 2.3.3 Interventions

The intervention measures of the treatment group are simple acupuncture combined with fluoxetine treatment (including needle material, treatment point selection, implementation technique, needle retention time, drug use, and treatment course), while the control group is treated with placebo acupuncture combined with fluoxetine, or fluoxetine alone.

#### 2.3.4 Outcome indicators

Main outcome: Hamilton Depression Scale (HAMD) decreased or HAMD reduction rate ≥25%, (reduction rate = (total score after treatment-total score before treatment)/total score before treatment × 100%).

Secondary outcome: Incidence rate of toxic and adverse reactions (nausea and vomiting, diarrhea, headache, and insomnia, etc.), Treatment Emergent Symptom Scale, Self-rating Depression Scale, and Activities of Daily living scale.

### 2.4 Exclusion criteria

1. Repeatedly published literature.
2. The data of the article is incomplete, so contact the literature which is still not available to the author.
3. Literature without relevant outcome indicators.

### 2.5 Search strategy

As of January 2021, it is searched in PubMed, Embase, the Cochrane Library, the web of science, China Biomedical Literature Database, China national knowledge infrastructure, Wanfang, and VP information Chinese Journal Service Platform databases. The search is conducted by the combination of subject words and free words. Chinese search words include “stroke,” “depression,” “acupuncture,” and “Fluoxetine,” while the English search words include “poststroke depression,” “Fluoxetine,” “acupuncture” and so on. Take PubMed as an example, the retrieval strategy is shown in Table 1.

**Table 1:** Retrieval strategy of PubMed.

Number	Search terms
#1	fluoxetine [MeSH]
#2	fluoxetine [Title/Abstract]
#3	#1OR#2
#4	acupuncture [MeSH]
#5	acupuncture[Title/Abstract]
#6	#4OR#5
#7	post-stroke depression [Title/Abstract]
#8	#3AND#6AND#7

### 2.6 Data screening and extraction

**Figure 1:**
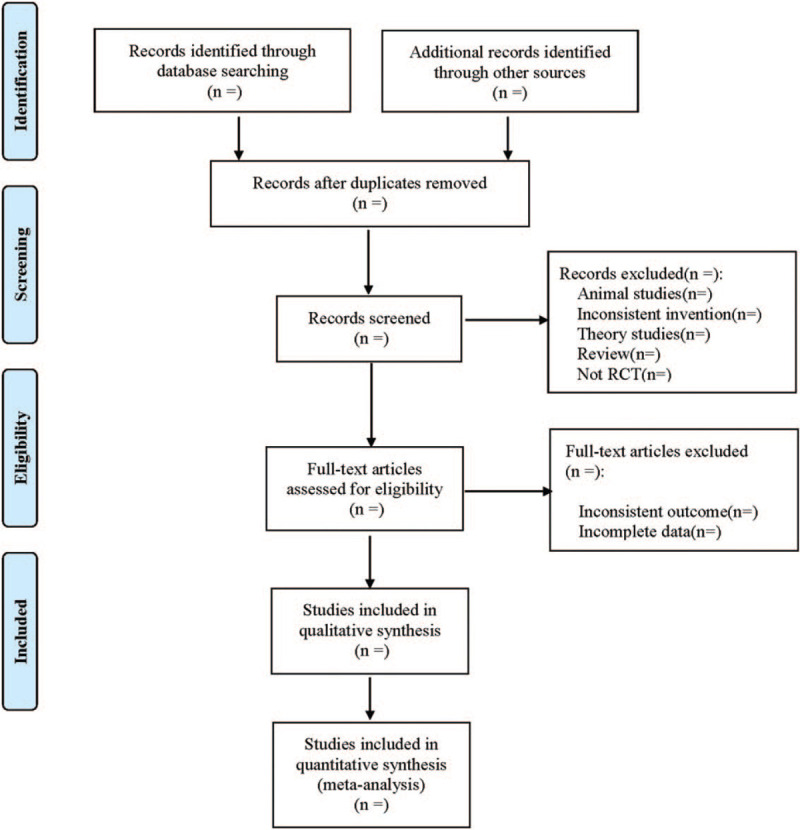
Flow diagram.

For all the retrieved literature, the EndnoteX9 software is included. Titles and abstracts are read by 2 independent researchers and excluded studies that do not meet the inclusion criteria. The researchers further read the full text of the remaining articles to determine whether they met the inclusion criteria. Then they cross-validate the conclusions. If the 2 researchers find it difficult to agree on whether a study should be included, they can resolve their differences by discussing a third party. The extracted data include the name of the first author, the year of publication, the number of cases in each group, intervention measures and control measures, outcome indicators and so on. The screening process is shown in Figure 1.

### 2.7 Literature quality evaluation

The 2 researchers independently evaluate the quality of the included trial. According to the bias risk tools described in the systematic Review Manual of Cochrane interventions, RCT is evaluated on 6 aspects: selection bias, implementation bias, measurement bias, follow-up bias, reporting bias, and other biases. The evaluation results are divided into: low risk, high risk, unclear. Cross-check, differences are resolved through discussions with third party personnel. Regardless of the quality of the literature, all studies that meet the inclusion criteria will be included. Although the evaluation results do not affect the screening results, the impact of research quality will be taken into account when interpreting the results.

### 2.8 Statistical analysis

#### 2.8.1 Data analysis and processing

The meta-analysis of the data is completed with the help of the software to RevMan5.3. In this study, the counting data are expressed by the risk ratio, the measurement data are expressed by the mean difference, and the 95% confidence interval is calculated. The heterogeneity test indexes are χ^2^ and *P*. In terms of heterogeneity, if *I*^*2*^* ≤* 50%*, P* *≥* .1, there is no heterogeneity, select the fixed effect model; if *I*^*2*^ *>* 50%*, P* *<* .1, indicate that there is heterogeneity, choose the random effect model. Inverted funnel chart analysis is used to test the existence of publication bias.

#### 2.8.2 Dealing with missing data

If there is missing data in the article, contact the author by phone or email and complete the data before analysis. If the author cannot be contacted, or if the author has lost the relevant data, no meta-analysis will be performed.

#### 2.8.3 Subgroup analysis

Subgroup analysis is carried out according to the age of middle-aged and elderly; subgroup analysis is carried out for male and female according to gender; subgroup analysis is carried out for cerebral hemorrhage and cerebral infarction according to different diseases; subgroup analysis is carried out according to the course of disease.

#### 2.8.4 Sensitivity analysis

A variety of factors will have an impact on the research results, through the selection of the literature of various factors are eliminated and compared one by one, in order to judge the stability of the results.

#### 2.8.5 Assessment of reporting biases

In order to know whether there is publication bias in the article, draw the funnel chart; if the funnel chart result is skewed distribution, it can be considered that there is a certain publication bias.

#### 2.8.6 Evidence quality evaluation

The quality of evidence is evaluated by Grading of Recommendation Assessment, Development and Evaluation score scale, which is divided into high, medium, low, and very low. It includes 5 aspects: bias risk, consistency, directness, accuracy, and publication bias.

## 3 Discussion

There is no clear conclusion on the mechanism of poststroke depression. At present, scholars at home and abroad mainly agree with 2 theories: biological mechanism and social and psychological mechanism. Biological mechanisms include neurotransmitters, inflammatory factors, neuroendocrine and stroke lesions. The abnormalities of neurotransmitters including 5-hydroxytryptamine (5-HT), norepinephrine and acetylcholine, and the increase of inflammatory factors including C-reactive protein and tumor necrosis factor-α, which further affect the abnormality of 5-HT. Neuroendocrine includes hypothalamus-pituitary-thyroid axis and hypothalamus-pituitary-suprarenal gland axis, which cause hormone abnormalities in the body, lead to hippocampal atrophy and induce depression.^[22]^ Fluoxetine, as the most widely used first-line antidepressant in the world, mainly acts on presynaptic nerve endings and inhibits reuptake after 5-HT release, thus increasing the level of available 5-HT in the brain and achieve therapeutic effect.^[23]^ Treatment time is recommended for 6 to 8 months or more.^[24]^

From the theoretical analysis of traditional Chinese medicine, poststroke depression is mainly due to emotional discomfort, liver loss and catharsis, spleen loss of health, heart loss of support, liver and kidney weakness finally lead to Zang-fu organs, Yin and Yang Qi and blood imbalance, the spirit has nothing to do with the disease. At present, acupuncture is mainly treated from the Governor Meridian. Hand and foot Jueyin Meridian and the hand Shaoyin Heart Meridian, and the acupoints are mainly Baihui, Neiguan, Shenmen, Taichong, and Hegu.^[25]^ Baihui belongs to the governor pulse, and the governor pulse belongs to the brain, which is the key point to regulate the function of the brain. Neiguan is the acupoint of the pericardial meridian, which is connected with the Sanjiao Meridian, which can widen the chest and smooth Qi, relieve depression and calm the mind. Shenmen is the acupoint of the hand Shaoyin Heart Meridian, which can calm the mind. Hegu and Taichong are called Siguan points, which can soothe the liver and relieve depression. Some experiments have shown that electroacupuncture Baihui, Shenmen and Taichong have an antidepressant effect, and its mechanism may be related to the inhibition of hyperfunction of the hypothalamus-pituitary-suprarenal gland axis.^[26]^ Some studies^[27]^ have shown that acupuncture can stimulate the cerebral cortex to increase the contents of NE, 5-HT and DA, inhibit the hypothalamic-pituitary-adrenocortical axis, improve and inhibit neuronal apoptosis, and thus improve the recovery of neuronal injury by Poststroke depression.

We hope to meta-analyze the existing randomized controlled trials of acupuncture combined with fluoxetine in the treatment of poststroke depression to evaluate the efficacy and safety of acupuncture combined with fluoxetine in the treatment of poststroke depression, so as to provide reliable evidence-based basis for clinical application. However, due to the small sample size and the low quality of random control, it is difficult to implement the blind acupuncture method, and the outcome index is incomplete; the therapeutic effect of acupuncture combined with fluoxetine in the treatment of poststroke depression still needs to further expand the sample size, improve the research quality, improve the outcome index, and actively explore effective scientific methods to evaluate the efficacy of acupuncture. Due to the limitation of language ability, we only search English and Chinese literature and may ignore studies or reports in other languages.

## Author contributions

**Data collection:** Peng Gong and Xiujuan Ma.

**Data curation:** Peng Gong, Xiujuan Ma.

**Funding acquisition:** Jianhao Bi.

**Funding support:** Jianhao Bi.

**Resources:** Peng Gong, Lijing Gao.

**Software:** Xiujuan Ma, Lijing Gao.

**Supervision:** Jianhao Bi.

**Writing – original draft:** Peng Gong, Xiujuan Ma.

**Writing – review & editing:** Peng Gong, Jianhao Bi.
